# A Single-Center Experience on Below-The-Knee Endovascular Treatment in Diabetic Patients

**Published:** 2020-02-20

**Authors:** E Dinoto, F Pecoraro, D Mirabella, F Ferlito, A Farina, N Lo Biundo, P Orlando-Conti, G Bajardi

**Affiliations:** 1 Vascular Surgery Unit “P. Giaccone” Hospital; 2 Department of Surgical, Oncological and Oral Sciences (Di.Chir.On.S.), University of Palermo; Palermo, Italy

**Keywords:** diabetic foot, ulcer, endovascular surgery, balloon angioplasty

## Abstract

Diabetic ulceration of the foot is a major global medical, social and economic problem and is the most frequent end-point of diabetic complications. A retrospective analysis from February 2017 to May 2019 of diabetic patients presenting below-the-knee artery disease (PAD) was carried out. Only patients treated with endovascular techniques as first choice treatment were evaluated. Outcome measured was perioperative mortality and morbidity. Freedom from occlusion, secondary patency and amputation rate were all registered. Additional maneuvers including stenting or angioplasty with drug eluting balloon (DEB) were reported. A total of 167 (101 male/66 female) patients with a mean age of 71 years were included in the study. A Rutherford 3, 4, 5 and 6 categories were reported in 5, 7, 110 and 45 patients, respectively. No perioperative mortality was reported. Morbidity occurred in 4 (4.4%) cases and consisted of pseudoaneurysm. Additional stenting during first procedure was required in 7 (4%) patients, drug eluting balloon was needed in 56 (33%) patients. At 1-year follow-up, estimated freedom from occlusion and secondary patency was 70% and 80% respectively. Major amputation rate was 2.4%, minor amputation rate was 41.9%. In our experience, extreme revascularization in search of distal direct flow reduce the rate of amputations with an increase in ulcer healing. New materials and techniques such as drug eluting technology, used properly, can improve outcome.

## I. INTRODUCTION

Diabetic ulceration of the foot is a major global medical, social and economic problem and is the most frequent major end-point of diabetic complications. Diabetic neuropathy and peripheral vascular disease are the main etiological factors in foot ulceration and may act alone, together, or in combination with other factors such as microvascular disease, biomechanical abnormalities, limited joint mobility and increased susceptibility to infection.1 Diabetic foot patients are among the most complex and vulnerable of all diabetes patients; between 0.03% and 1.5% of patients with diabetic foot require an amputation.^2^ After major lower amputation the cumulative mortality rate at 1 year was 30.7%.^3^

## II. METHODS

A retrospective analysis from February 2017 to May 2019 of diabetic patients presenting below the knee artery disease (PAD) and diabetic foot was carried out. Patients treated with endovascular technique as first choice treatment during the observation period were included in the study. Simple PTA was performed in every procedure. Measured outcomes were perioperative mortality and morbidity. Freedom-from-occlusion, secondary patency and amputation rate were all registered. Additional maneuvers including stenting or angioplasty with drug eluting balloon (DEB) were reported.

## III. RESULTS

A total of 167 (101 male//66 female) patients with a mean age of 71 years were included in the study. A Rutherford 3, 4, 5 and 6 class was reported in 5, 7, 110 and 45 patients, respectively. Ulcers were identified according to Texas classification (grade I 48%, grade II 33%, grade III 19%). No perioperative mortality was reported. Morbidity occurred in 4 (4,4%) cases and consisted of pseudoaneurysm. Additional stenting during first procedure was required in 7 (4%) patients and drug eluting balloon in 56 (33%) patients. Indication for stenting was arterial dissection; Drug eluting balloon was used for residual stenosis or recoil after first PTA. The mean follow-up was 12 months. At 1-year follow-up, estimated freedom-from-occlusion and secondary patency was 70% and 80%, respectively; major amputation rate was 2.4%, minor amputation rate was 41.9%.

## IV. DISCUSSION

Ulceration rarely results from a single pathology. The etiopathogenesis of diabetic foot disease is multifactorial encompassing three main factors namely, neuropathy, ischaemia and infection leading to tissue necrosis and ulcer formation. Other factors are foot biomechanics and weight bearing, peripheral vessel calcification, trauma, diabetic autonomic neuropathy and microangiopathy and diabetic skeletal disease.^1^,^4^ It is the interaction of these contributory causes which lead to the breakdown of the foot at risk.^5^ Thus Diabetic foot disease is an important problem challenging diabetologists, internists and surgeons who, in specialized diabetic foot clinics, should work together to coordinate revascularization procedures to aggressively treat infections and to manage medical comorbidities in a multidisciplinary forum.

The role of the vascular surgeon in this pattern of events is to cooperate with in order to get the best outcome from revascularization procedures. The aim of such interventions is to increase the blood flow to the foot which in turn enhances cutaneous oxygen pressure promoting infection clearance and ulcer granulation at a crucial time point.^6^ Techniques such as subintimal angioplasty of the femoral-popliteal artery segment, retrograde angioplasty using trans-pedal access and arterial flossing with anterograde-retrograde intervention are several technical innovations that improve the percutaneous transluminal angioplasty success rate in the diabetic limb which, as open techniques, have the final outcome of increasing distal vascularization. Several studies have highlighted the importance of a deep revascularization in view of the fact that below the knee revascularization reduces the rate of non-healing, minor and major amputations.^7^ Endovascular therapy plays a major role in the treatment of critical limb ischemia with below the knee arteries involved. New materials, unconventional techniques and the operator’s skill can improve the outcome. An important part is also played by devices like drug eluting balloons (DEB). In literature there are few studies on the advantages of using DEB in this district^8^,^9^ but in our experience a secondary Drug Coated balloon angioplasty, during first or following approach offers the advantages of simplicity and reduction in the need for stenting while increasing primary and secondary patency rates.^10^

Another significant factor is the partnership between the different specialists such as diabetologist, cardiologist, general surgeon, plastic surgeon and others. This natural passage that complements the skills and knowledge of each attending physician would increase successful limb salvage and functional outcomes. The American Diabetes Association recommends, as well, a multidisciplinary approach for individuals with foot ulcers and those who are at high-risk of foot loss. In particular, the presence of a multidisciplinary team aimed at preventive care has been reported to decrease the risks associated with diabetic foot and amputation by 50% to 85%.^11^

## V. CONCLUSION

Extreme revascularization in search of distal direct flow reduces the rate of amputation and increases ulcer healing. The literature shows better outcomes, limb salvage and rate amputation reduction when a vascular surgeon is part of a multi-disciplinary treatment team.^12^ New materials, used properly, can improve the outcomes.
